# Early treatment failure during treatment of *Plasmodium falciparum* malaria with atovaquone-proguanil in the Republic of Ivory Coast

**DOI:** 10.1186/1475-2875-11-146

**Published:** 2012-05-02

**Authors:** Nathalie Wurtz, Aurélie Pascual, Adeline Marin-Jauffre, Housem Bouchiba, Nicolas Benoit, Marc Desbordes, Maryse Martelloni, Vincent Pommier de Santi, Georges Richa, Nicolas Taudon, Bruno Pradines, Sébastien Briolant

**Affiliations:** 1 Unité de Parasitologie - Unité de Recherche pour les Maladies Infectieuses et Tropicales Emergentes - UMR 6236, Institut de Recherche Biomédicale des Armées, Marseille, France; 2 Centre National de Référence du Paludisme, Marseille, France; 3 UMR MD3 Infections Parasitaires: Transmission, Physiopathologie et Thérapeutique, Aix-Marseille Université, Institut de Recherche Biomédicale des Armées, Marseille, France; 4 Centre d’Epidémiologie et de Santé Publique des Armées, Marseille, France; 5 Centre médical des armées de Nîmes Orange Laudun, antenne colonel De Chabrières, Nîmes, France

**Keywords:** Malaria, Plasmodium falciparum, Malarone®, Atovaquone-proguanil, Cytochrome b, Resistance, Clinical failure, in vitro, Anti-malarial drug

## Abstract

The increased spread of drug-resistant malaria highlights the need for alternative drugs for treatment and chemoprophylaxis. The combination of atovaquone‐proguanil (Malarone®) has shown high efficacy against *Plasmodium falciparum* with only mild side-effects. Treatment failures have been attributed to suboptimal dosages or to parasite resistance resulting from a point mutation in the *cytochrome b* gene. In this paper, a case of early treatment failure was reported in a patient treated with atovaquone-proguanil; this failure was not associated with a mutation in the parasite *cytochrome b* gene, with impaired drug bioavailability, or with re-infection.

## Background

Increasing reports of drug-resistant *Plasmodium falciparum* throughout the world have forced changes in both prevention and treatment. Atovaquone-proguanil (A-P, Malarone®, GlaxoSmithKline Inc) is one of the common first-line agent for the prophylaxis [1-3] and treatment [2,4,5] of *falciparum* malaria in France and causes only mild side-effects. Since the introduction of the A-P combination, several cases of treatment failure have been observed in travellers returning from Africa [6-14]. Treatment failures have been attributed to suboptimal dosage or impaired bioavailability, re-infection or to a point mutation in the *cytochrome b* gene (*pfcytb*) [4,9,15]. In this paper, a case of A-P treatment failure in a military employee stationed in the Republic of Ivory Coast was reported; this treatment failure was not due to low plasma levels of the drug, mutations in the *pfcytb* gene, or re-infection.

## Case presentation

The patient was a 45-year-old military employee deployed to Abidjan in the Republic of Ivory Coast (Port Bouet camp). He had been stationed in Abidjan for six months and had repeatedly forgotten to take the prescribed anti-malarial chemoprophylaxis drug, doxycycline. He presented general malaise and headaches on 27 September, 2011, and was treated by self-medication with paracetamol. Two days later (29 September, 2011 (day 0)), the patient consulted the medical centre of the camp, where he presented headache, myalgias, chills and fever with a temperature of 39.7°C. A blood smear examination revealed *P. falciparum* at a parasitaemia of 0.74%, and a rapid diagnosis test confirmed *Plasmodium* infection. The patient was hospitalized and immediately treated with the standard treatment of Malarone®, four 250 mg tablets daily on day 0, day 1 and day 2, associated with paracetamol and the continuation of chemoprophylaxis with doxycycline. The tablets were taken with fatty food and were well tolerated. No vomiting or diarrhoea occurred during hospitalization. At day 1, the patient was asymptomatic and afebrile. At day 2, after the last intake of Malarone®, the patient experienced a febrile peak at 38.5°C and blood smear examination revealed a parasitaemia of 1.3%. Because of the recrudescent parasitaemia and according to WHO guidelines for the assessment and monitoring of anti-malarial drug efficacy for the treatment of uncomplicated *falciparum* malaria [16], the possibility of early A-P treatment failure was considered, and the patient was treated with 625 mg of quinine base (Quinimax®) orally three times daily for seven days, doxycycline (100 mg twice daily for seven days) and paracetamol. On 4 October (day 5), the parasitaemia decreased to 0.02%, and on 7 October (day 8), a QBC test was negative.

## Methods

Whole blood specimens from the first (day 0) and second (day 2) episodes of malaria were submitted to the reference laboratory for gene amplification by polymerase chain reaction (PCR), sequencing, genetic analysis and quantification of the plasma concentrations of drugs. No *in vitro* assays of the *P. falciparum* isolates could be performed. The DNA of both samples was extracted from blood samples using the QIAamp DNA Mini Kit according to the manufacturer’s recommendations (Qiagen, France). Confirmation of *P. falciparum* mono-infection was performed by real-time LightCycler® PCR (Roche, Meylan, France), as described elsewhere [17]. The *pfcytb* and dihydrofolate reductase (*pfdhfr*) genes were amplified by PCR and sequenced for both isolates to detect mutations associated with resistance to A-P, respectively, as described [15,18,19]. Molecular markers of resistance [19], such as *pfcrt* (chloroquine resistance transporter), *pfmdr1* (multidrug resistance 1 protein), *pfnhe1* (Na^+^/H^+^ exporter 1), *pfdhps* (dihydropteroate synthetase), *pftetQ* (tetQ family GTPase) and *pfmdt* (metabolite/drug transporter) were also assessed. The single-nucleotide polymorphism and copy number assays for these different genes were performed as previously described [15,20-22]. The parasite diversity between day 0 and day 2 was determined by genotyping the TRAP, 7A11, C4M79, Pf2802, and Pf2689 microsatellite loci; the highly polymorphic loci of the merozoite surface protein 1 and 2 antigen genes (*msp1msp2*); and the highly polymorphic loci of the glutamate-rich protein gene (*glurp*) using fluorescent end-labelled nested PCR and restriction fragment length polymorphism analysis. The primer sequences, PCR conditions, and genotyping methods have been described elsewhere [23-25]. The drug absorption and compliance were estimated by quantification of the drug levels in the patient’s plasma; these assays were performed using a Waters Acquity UPLC instrument (Milford, MA, USA). Separation was carried out on an Acquity BEH C8 column (50 mm × 2.1 mm, 1.7 μm) maintained at 40°C. The mobile phase consisted of solvent A (0.5% acetic acid in purified water) and solvent B (acetonitrile). Two gradient programmes were used, one for the quantification of proguanil, cycloguanil and doxycycline and the second for the quantification of atovaquone. The flow rate was 0.8 mL/min. The injection volume and total run time were, respectively, 5 μL and 3 min. A purification step was performed before analysis using i) an OASIS® HLB SPE cartridge (Waters, Milford, MA, USA) for proguanil, cycloguanil and doxycycline and ii) protein precipitation by the addition of two volumes of an ACN/H_2_O/acetic acid solution (85:14:1, v/v/v).

## Consent

Informed consent was not required because the sampling procedures and testing are part of the French national recommendations for the care and surveillance of malaria.

## Results

As previously observed on the blood smear, *P. falciparum* is the only species detected by real-time PCR. The whole sequencing of the *pfcytb* gene, which encodes the target of atovaquone, revealed a wild-type *P. falciparum* isolate on day 0 and day 2 (M133, Y268). Moreover, no other point mutation was identified in the *pfcytb* gene. Genotyping of the whole *pfdhfr* gene showed that the three of the five major mutations (A16, N51I, C59R, S108N, I164) associated with proguanil (cycloguanil)/ pyrimethamine resistance were found in isolates from day 0 and day 2. Genotyping of the *pfcrt* gene (wild type, K76), the *pfmdr1* gene (N86, Y184F, S1034, N1042 and D1246) and the *pfdhps* gene (S436A, A437G, K540, A581, and A613) showed identical alleles. Only one copy each of *pfmdr1*, *pftetQ* and *pfmdt* was found in each sample. The *pfnhe1* microsatellite ms4760 exhibited profile 22 with one DNNND repeat and two DDDNHNDNHNN repeats. Genotyping of the parasites from days 0 and 2 using the microsatellite loci, *msp1*, *msp2*, and *glurp* showed that the alleles were identical for each locus in the two samples.

Ultra high-performance liquid chromatography was performed on a blood sample withdrawn just before the third Malarone® administration. Thus, the results showed the residual levels of drugs just before the last intake. The atovaquone plasma concentration was 5.1 μg/mL, and the proguanil and doxycycline levels were, respectively, equal to 380 ng/mL and 509 ng/mL. The plasma concentration of cycloguanil was not determined due to the presence of an interfering signal.

## Conclusion

Malarone®, a fixed-dose combination of A-P, is highly effective for the treatment and prophylaxis of multi-drug-resistant *falciparum* malaria [1-5], and it is a useful agent due to its convenient mode of administration (oral), short treatment course (three days) and limited side effects. Atovaquone, a ubiquinone analogue that binds to CYTB of plasmodial mitochondria, exerts its action by inhibiting electron transfer in the respiratory chain [26,27]. The proguanil metabolite cycloguanil acts by inhibiting the parasite PfDHFR protein, which is involved in pyrimidine biosynthesis, and the addition of proguanil leads to an enhancement of atovaquone’s activity and reduces the chance of mutations arising in the mitochondrial DNA of the malaria parasite [28,29]. Since the introduction of the A-P combination, few cases of treatment failure have been identified in travellers returning from Africa [6-14]. Treatment failures have been generally attributed to suboptimal dosage, re-infections with a new parasite, or to a point mutation (Y268N, Y268S or Y268C) in the *pfcytb* gene [4,9,14,18]. However, several cases of clinical treatment failure were not associated with any known *pfcytb* mutation, the plasma drug concentrations were well within curative range, and re-infection was excluded [7,8,13]. In this paper, a case of early A-P treatment failure not associated with *pfcytb* mutations in a military employee stationed in Abidjan in the Republic of Ivory Coast was reported.

The residual drug levels were quantified on a sample withdrawn between 23 to 24 hours after the second Malarone® intake. No recommended therapeutic concentrations are available for the A-P. Association and data are missing especially in the treatment of uncomplicated *falciparum* imported malaria in Caucasians. However, regarding pharmacokinetics parameters of the both drugs, and more particularly the elimination half-life, the maximum concentrations and steady-state concentrations, impaired bioavailability can be excluded [30-33]. Data about the relationship between plasma doxycycline concentration and time in the same population are also lacking. However, drug plasmatic level is in the same other as that expected [34,35].

For further analysis, the day 0 and day 2 *P. falciparum* isolates were investigated for point mutations in the *pfcytb* codon 268 [15], and this analysis revealed wild-type alleles in both isolates. Genotyping of the blood samples from day 0 and day 2 at microsatellite loci (TRAP, 7A11, C4M79, Pf2802, and Pf2689) and at the highly polymorphic loci of the *msp1* and *msp2* antigen genes and of the *glurp* gene were performed. The results showed that the two samples had the same molecular signature and complete homology, excluding the possibility of a re-infection, and these results were confirmed by genotypic analysis of resistance markers. Based on these results, the clinical and parasitological features of the patient between day 0 and day 2, and the WHO definition of treatment failure [16,36], this case could be classified as an early treatment failure.

Several other factors may also contribute to the emergence of A-P resistance, including hyperparasitaemia, rapid metabolism of proguanil or prior exposure to related drugs [37]. In this case, high parasitaemia was not observed (parasitaemia on day 0 = 0.74%; parasitaemia on day 2 = 1.3%). Caucasians are known to metabolize proguanil to cycloguanil relatively rapidly compared to other ethnic groups, leaving the parasites exposed to atovaquone alone for a longer period of time [31]. However, there was no clear evidence to implicate this mechanism as a factor in the emergence of A-P resistance in this case. Moreover, the metabolic status (i.e. poor/extensive) of the patient can’t be assumed with this only concentration versus time level. In the context of this case report, the genotype or an individual pharmacokinetics for proguanil was not possible.

In summary, this case represents the first observation of the clinical failure of A-P treatment for *P. falciparum* infection in a military in Ivory Coast that was not due to impaired drug bioavailability, resistance due to *pfcytb* mutations or re-infection with a new parasite. The absence of mutations in *pfcytb* suggests that alternative mechanisms may be involved in the resistance to this drug combination [7,8]. Indeed, resistance may be associated with either inhibition or alteration of key enzymes that are targets for anti-malarial drugs, or alteration of drug accumulation in the parasite that results from reduced uptake of the drug, increased efflux, or a combination of both processes [19].

Although clinical failure of A-P treatment is rare among travellers, increased vigilance is required during treatment and post-treatment, and the monitoring of the parasite population should be strengthened. Further research is required to gain a better understanding of the mechanisms involved in the clinical failure observed after treatment with Malarone®, one of the few available drugs used to treat infections with multidrug-resistant *P. falciparum* parasites.

## Competing interests

The authors declare that they have no competing interests.

## Authors’ contributions

GR, MD and VPS carried out diagnostic monitoring of the patient, collection of clinical and epidemiological data. NW, AP, AJ, HB and NB carried out the molecular genetic studies. NT and MM performed the quantification of plasmatic concentration of atovaquone-proguanil and cycloguanil. BP and SB conceived and coordinated the study. NW, SB, NT and BP drafted the manuscript. All authors read and approved the final manuscript.
